# All reported non-canonical splice site variants in *GLA* cause aberrant splicing

**DOI:** 10.1007/s10157-023-02361-x

**Published:** 2023-05-31

**Authors:** Eri Okada, Tomoko Horinouchi, Tomohiko Yamamura, Yuya Aoto, Ryota Suzuki, Yuta Ichikawa, Yu Tanaka, Chika Masuda, Hideaki Kitakado, Atsushi Kondo, Nana Sakakibara, Shinya Ishiko, China Nagano, Shingo Ishimori, Joichi Usui, Kunihiro Yamagata, Masafumi Matsuo, Kandai Nozu

**Affiliations:** 1 Department of Pediatrics, Kobe University Graduate School of Medicine, 7-5-1 Kusunoki-Cho, Chuo-Ku, Kobe, Hyogo 650-0017 Japan; 2 Department of Nephrology, Faculty of Medicine, University of Tsukuba, Tsukuba, Japan; 3 Department of Pediatrics, Hokkaido University Graduate School of Medicine, Sapporo, Japan; 4 Department of Physical Rehabilitation and Research Center for Locomotion Biology, Kobe Gakuin University, Hyogo, Japan

**Keywords:** GLA, Fabry disease, Minigene assay, Aberrant splicing, Deep intronic variant, Inherited kidney disease

## Abstract

**Background:**

Fabry disease is an X-linked lysosomal storage disorder caused by insufficient α-galactosidase A (GLA) activity resulting from variants in the *GLA* gene, which leads to glycosphingolipid accumulation and life-threatening, multi-organ complications. Approximately 50 variants have been reported that cause splicing abnormalities in *GLA*. Most were found within canonical splice sites, which are highly conserved GT and AG splice acceptor and donor dinucleotides, whereas one-third were located outside canonical splice sites, making it difficult to interpret their pathogenicity. In this study, we aimed to investigate the genetic pathogenicity of variants located in non-canonical splice sites within the *GLA* gene.

**Methods:**

13 variants, including four deep intronic variants, were selected from the Human Gene Variant Database Professional. We performed an *in vitro* splicing assay to identify splicing abnormalities in the variants.

**Results:**

All candidate non-canonical splice site variants in *GLA* caused aberrant splicing. Additionally, all but one variant was protein-truncating. The four deep intronic variants generated abnormal transcripts, including a cryptic exon, as well as normal transcripts, with the proportion of each differing in a cell-specific manner.

**Conclusions:**

Validation of splicing effects using an *in vitro* splicing assay is useful for confirming pathogenicity and determining associations with clinical phenotypes.

**Supplementary Information:**

The online version contains supplementary material available at 10.1007/s10157-023-02361-x.

## Introduction

Fabry disease (OMIM no. 301500) is an X-linked lysosomal storage disorder resulting from insufficient α-galactosidase A (GLA; GenBank accession no. 2717) activity caused by mutations in the *GLA* gene at Xq22. An enzymatic defect in GLA protein results in the accumulation of globotriaosylceramide and related glycosphingolipids throughout the body [1, 2]. Typically, hemizygous males with little (< 1% of normal) or no GLA activity suffer from characteristic abnormalities in multiple organs, namely neurological (acroparesthesia), cutaneous (angiokeratomas), renal (proteinuria, kidney failure), cardiovascular (cardiomyopathy, arrhythmia), and cerebrovascular (stroke) symptoms. This phenotype is known as the "classic type". In contrast, some patients have a somewhat preserved level of enzyme activity, and only display predominant cardiac or renal symptoms later in life; these phenotypes are sub-classified as the “cardiac type” [3, 4] and “renal type” [5], respectively.

To date, 1086 variants of the *GLA* gene have been registered in the Human Gene Mutation Database (HGMD) Professional (released in 2021.2) (https://portal.biobase-international.com/hgmd/pro/start.php). Among these, approximately 5% correspond to splicing changes that may cause aberrant splicing. Most intronic variants of the *GLA* gene registered in HGMD are located at canonical splice sites (dinucleotides at each end of the intron, usually GT at the 5′-end and AG at the 3′-end). Generally, these variants within canonical splice sites cause aberrant splicing because they impact highly conserved sequences that determine exon–intron boundaries [6]. Although several reported variants are located outside these canonical splice sites, their pathogenicity is not yet confirmed.

Recently, various bioinformatics tools have been developed to predict the possible pathogenic effects of missense and intronic variants [6, 7]; however, these predictions are not always correct. Presently, transcriptional analysis of patient samples is the most appropriate method for identifying splicing aberrations. However, RNA from affected organs is not always available. Moreover, it is often difficult to analyze mRNA obtained from peripheral leukocytes owing to low content and fragility. As an alternative, minigene splicing analyses have been developed [8–14]. In this study, we performed a functional minigene splicing assay on the reported *GLA* non-canonical intronic variants to examine possible splicing defects.

## Materials and methods

### Variant nomenclature and selection of non-canonical splice site variants

**Fig. 1:**
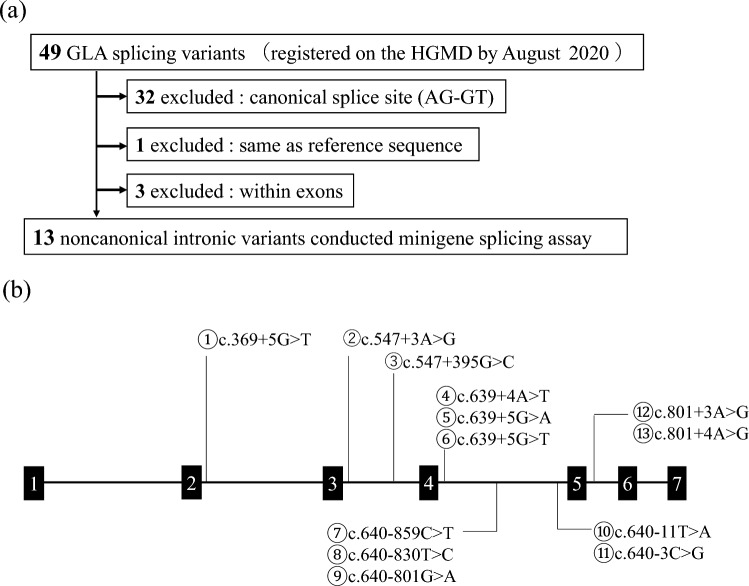
**a** Selection of the candidate variants. **b** Location of each variant in the *GLA* gene

*GLA* variants were numbered according to the guidelines of the Human Genome Variation Society (https://www.hgvs.org/mutnomen) using the NCBI Reference Sequence NM_000169.3. Candidate variants for minigene analysis were selected from HGMD Professional. By August 2020, 49 variants had been identified as splicing substitutions that caused splicing abnormalities. Among these, 32 variants were located at canonical splice sites, while three exonic variants were excluded. In addition, one variant was excluded because of suspected erroneous registration of the its information in the HGMD database. Therefore, we analyzed the remaining 13 *GLA* intronic variants (Fig. 1a). Among these, nine variants were adjacent to exons that did not contain AG-GT canonical splice sites, and the other four were located more than 100 nucleotides away from the nearest exon–intron junction, typically referred to as deep introns (Fig. 1b). To prove that minigene analysis had accurately detected splicing aberrations, the intronic variant (c.640-16A>G), considered a single-nucleotide polymorphism (SNP), was analyzed as a negative control. The allele frequency of the variant in different population databases is 13.9% (gnomAD), 16.1% (1000G), and 7.7% (HGVD).

### Bioinformatics analysis

To predict potential alterations in the donor/acceptor splice sites, we used the following tools: SpliceSiteFinder-like (https://www.interactive-biosoftware.com), MaxEntScan (https://genes.mit.edu/burgelab/maxent/Xmaxentscan_scoreseq.html), and NNSPLICE (https://www.fruitfly.org/seq_tools/splice.html) via Alamut software v.2.11 (Interactive Biosoftware, Rouen, France; https://www.interactive-biosoftware.com). The default settings were used for all predictions. Each tool was assumed to predict altered splicing when the change in splice site score was ≥ 10% (MaxEntScan) or ≥ 5% (SpliceSiteFinder-like and NNSPLICE) [15, 16]. The splicing process is regulated by splicing regulatory elements (SREs) that recruit various RNA-binding proteins [17]. SREs include intronic and exonic splicing enhancers (ISEs and ESEs) and silencers (ISSs and ESSs). Variants that alter the composition, affinity, or function of spliceosomes can lead to improper identification of exon–intron boundaries, thereby causing aberrant splicing [18]. To predict alterations in SREs, we used ESE-finder (https://exon.cshl.edu/ESE/) and RESCUE-ESE (https://hollywood.mit.edu/burgelab/rescue-ese/) within the Alamut software v.2.11 as well as HumanSplicingFinder (https://umd.be/Redirect.html).

### Cell culture and minigene constructs

HEK293T and HeLa cells were cultured in DMEM and EMEM, respectively, with both media containing 10% fetal bovine serum and 1% penicillin–streptomycin (Fujifilm, Osaka, Japan). Wild-type genomic DNA fragments, including the targeted exon(s) and approximately 200 bp of surrounding introns, were amplified using Gflex DNA polymerase (Takara Bio, Shiga, Japan). Amplicons were subsequently cloned into the H492 vector based on the pcDNA3.0 mammalian expression vector (Invitrogen, Carlsbad, CA, USA) using the In-Fusion HD Cloning Kit (Takara Bio), according to the manufacturer’s instructions. Mutant plasmids were created via site-directed mutagenesis with the Prime STAR Mutagenesis Basal Kit (Takara Bio). The primer sequences used are listed in Supplementary Table S1. For deep intronic mutations (no. 7–9), cloning fragments were made via overlap extension polymerase chain reaction (PCR), followed by traditional restriction cloning using *NheI* and *BamHI* sites ligated with the DNA Ligation Kit Ver. 2.1 (Takara Bio), as described in Supplementary Fig. S1. Hybrid minigenes were transfected into HEK293T and HeLa cells using the Lipofectamine 3000 Transfection Kit (Thermo Fisher Scientific, Waltham, MA, USA). After 24 h, the total RNA was extracted from the cells and purified using the RNeasy Plus Mini Kit (QIAGEN, Hilden, Germany). Total RNA was reverse-transcribed into cDNA using the EcoDry Premix (Double Primed; Takara Bio) and then subjected to PCR amplification using gene-specific primers YH307 and YH308 (Supplementary Table S1). The PCR products were analyzed using an Agilent 2100 Bioanalyzer system (Agilent Technologies, Santa Clara, CA, USA).

### Data collection for transcriptome analysis of variant no. 9 (c. 640-801G>A)

Informed consent was obtained from the patient harboring mutation no. 9 (c.640-80G>A), whose blood sample was used for direct RNA sequencing. Total RNA was extracted using the RiboPure Blood Kit (Invitrogen) and an RNA stabilization agent (RNAlater; Invitrogen), after which a library was prepared using the SureSelect^XT^ RNA Direct Reagent Kit (Agilent Technologies). Briefly, mRNA obtained from the patient’s blood sample was randomly fragmented, and adaptor-ligated cDNA was synthesized using the SureSelect RNA Library Prep Kit, followed by PCR amplification to prepare cDNA library amplicons. The SureSelect RNA Target Enrichment Probe Library and cDNA library amplicons were hybridized using SureSelect^XT^ reagents and subjected to high-throughput sequencing using the MiSeq platform, as described in the Illumina manual (San Diego, CA, USA). The sequencing data were fed into the Strand NGS 4.0 software (Strand Life Sciences Pvt. Ltd., Bangalore, India) and visualized using the Integrative Genomics Viewer software (version 2.11.7) [19]. The cryptic exon inclusion ratio, or “percent spliced in” (PSI), describes the ratio of the cryptically spliced transcripts to the sum of both the cryptically and normally spliced transcripts. PSI was calculated according to the formula described by Sakaguchi et al. [20]. The PSI values ranged from 0 (completely skipped) to 1 (complete inclusion).

## Results

### Clinical phenotype

Although the clinical phenotype and enzymatic activity were not described for variant nos. 1 and 8, phenotypes were reported for the remaining 11 variants: nos. 2, 3, 4, 6, and 10–13 manifested as the classic type, whereas nos. 5, 7, and 8 were associated with later-onset or cardiac type. Variant no. 9 (c. 640-801G>A, often described as “IVS4+919G>A” or “c.639+919G>A”) was also identified in our genetic analysis cohort. The patient was an 81-year-old Japanese male who suffered from congestive heart failure. Neither detailed clinical information nor residual enzymatic activity was available, and the patient did not undergo dialysis treatment.

### Minigene splicing assay results

**Fig. 2:**
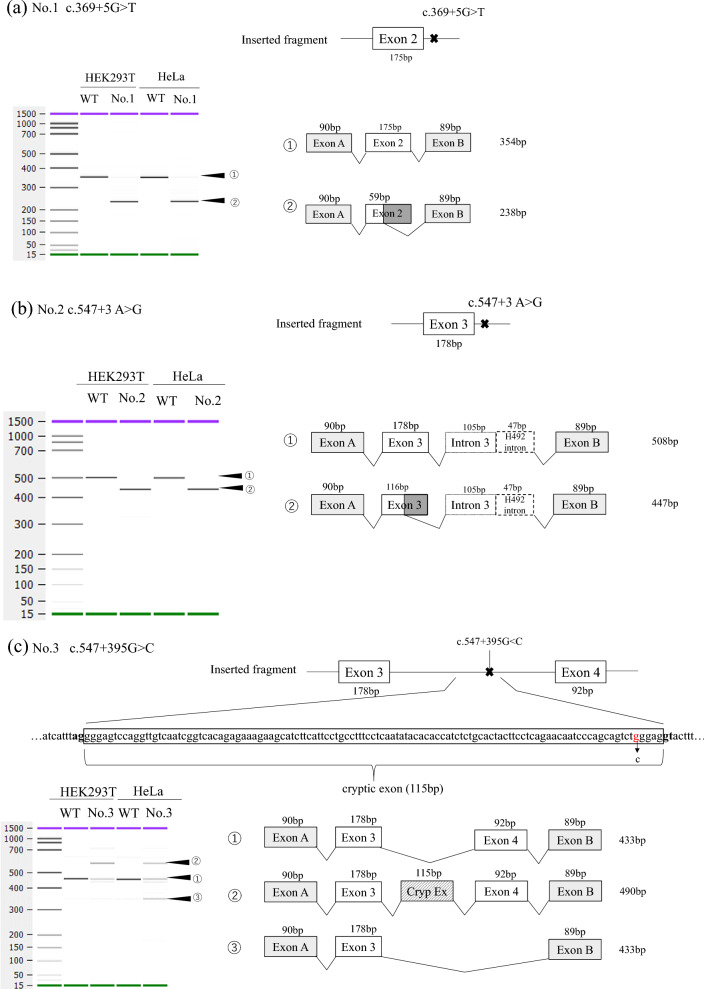

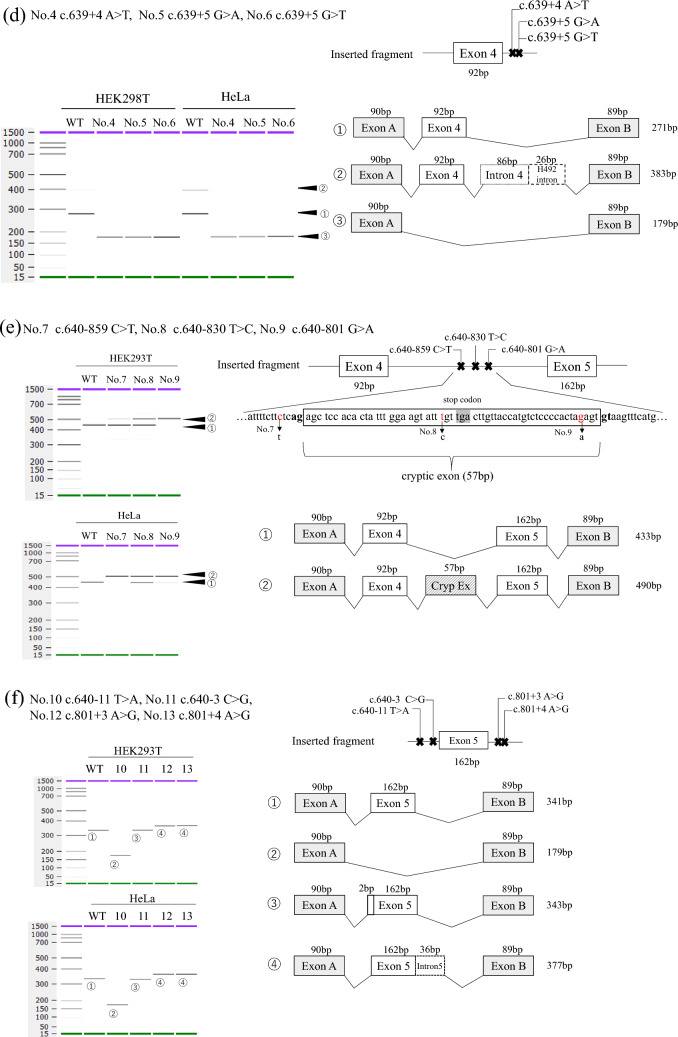
Results of transcriptional analysis using the splicing minigene assay. The left part shows the electropherogram obtained with the bioanalyzer. The upper right part shows each inserted fragment constructed with individual exons and flanking introns. The lower right part shows a schematic representation of the splicing outcome **a** No. 1 c.369+5G>T. **b** No. 2 c.547+3A>G. **c** No. 3 c.547+395G>C. **d** No. 4 c.639+4A>T, no. 5 c.639+5G>A, and no. 6 c.639+5G>T. **e** No. 7 c.640–859 C>T, no. 8 c.640–830T>C, and no. 9 c.640–801 G>A. **f** No. 10 c.640–11T>A, no. 11 c.640–3 C>G, no. 12 c.801+3 A>G, and no. 13 c.801+4 A>G

The minigene splicing assay revealed that all reported non-canonical intronic variants of *GLA* were associated with aberrant splicing (Table 1, Fig. 2, and Supplementary Fig. S2). We identified a partial deletion in the adjacent exon (nos. 1 and 2), exon skipping (os. 4–6, and 10), and partial inclusion of an adjacent intron (nos. 11–13). In addition, all deep intronic variants (os. 3–6) resulted in the creation of a cryptic exon. Regarding the protein effect, variant no. 10 exhibited a non-truncating defect (exon 10 consisted of 162 bp, a multiple of three), whereas the remaining variants caused truncating defects. Variant c.640-16A>G, considered to be an SNP, showed normal splicing (Supplementary Fig. S3).

**Table 1:** Phenotype and results of RT-PCR and minigene assay of the candidate variants

No	variant	Intron	References	Phenotype (hemizygous)	α-Gal A activity (normal range)	RT-PCR analysis	Minigene assay		
							Splicing outcome	RNA effect	Protein effect
1	c.369+5 G>T	2	[43]	ND	ND	ND	Partial deletion of exon 2 (116 bp)	r.254_369del	p.Tyr86Serfs*16
2	c.547+3 A>G	3	[23]	Classic	1.7 nmol/mg/h (17.6–57.6)	ND	Partial deletion of exon 3 (62 bp)	r.486_547+1del	p.Gly163Leufs*2
3	c.547+395G>C	3	[31]	Classic	5.06 nmol/mg/h (169.5–212.7)	Cryptic exon (115 bp)	• Cryptic exon (115 bp) • Normal transcripts	r.547_548ins115	p.Tyr184Glufs*39
4	c.639+4 A>T	4	[24]	Classic	1.0 U/mL (no data)	ND	Skipping of exon 4 (92 bp)	r.548_639del	p.Gly183Alafs*18
5	c.639+5 G>A	4	[26]	(Later-onset*)	ND	ND	Skipping of exon 4 (92 bp)	r.548_639del	p.Gly183Alafs*18
6	c.639+5 G>T	4	[25]	Classic	0.2 nmol/h/mL (> 0.3)	ND	Skipping of exon 4 (92 bp)	r.548_639del	p.Gly183Alafs*18
7	c.640–859 C>T	4	[32]	Classic	0.5 nmol/mg/h	Cryptic exon (57 bp)	• Cryptic exon (57 bp) • Normal transcripts	r.639_640ins57	p.Lys213_Pro214ins10*
					(25–75)				
8	c.640–830T>C	4	[44]	ND	ND	ND	• Cryptic exon (57 bp) • Normal transcripts	r.639_640ins57	p.Lys213_Pro214ins10*
9	c.640–801 G>A	4	[33]	Cardiac*	8.7 U/mg	Cryptic exon (57 bp)	• Cryptic exon (57 bp) • Normal transcripts	r.639_640ins57	p.Lys213_Pro214ins10*
					(9.1% of normal)				
10	c.640–11T>A	4	[27]	Classic*	0.02% of normal	Skipping of exon 5 (162 bp)	skipping of exon 5 (162 bp)	r.640_801del	p.Pro214_Met267del
11	c.640–3 C>G	4	[29]	Classic#	ND	ND	Partial inclusion of intron 4 (2 bp)	r.640-1_640ins2	p.Pro214Serfs*27
12	c.801+3 A>G	5	[30]	Classic*	0.5 (no unit description)	• Partial inclusion of intron 5 (66 bp) • Entire intron 5 (217 bp)	Partial inclusion of intron 5 (36 bp)	r.801_802ins36	p.Met267_Leu268ins4*
13	c.801+4 A>G	5	[30]	Classic*	ND	ND	Partial inclusion of intron 5 (36 bp)	r.801_802ins36	p.Met267_Leu268ins4*

*ND* not described

*Sex unknown

^#^Female

### Interpretation of variant pathogenicity and bioinformatics analysis for splicing defects

All variants were interpreted as having uncertain significance according to the ACMG guidelines [21] (Supplementary Table S2). Each in silico prediction tool for splice site score (SSF-like, MaxEntScan, and NNSPLICE) correctly predicted splicing defects for variants near splice sites (nos. 1, 2, 4–6, and 10–13). For deep intronic variants (nos. 3 and 7–9), splicing abnormalities could not be predicted by the tools that calculated changes in splice site score; however, except for variant no. 7, the in silico tools that predicted splicing defects based on alterations in SREs were effective for the deep intronic variants (Supplementary Table S3).

### Direct RNA sequencing for variant no. 9 (c.640-801G>A)

The cryptic exon inclusion ratio (PSI) for the patient harboring variant no. 9 was 0.42, whereas that of the control was 0.027 (Supplementary Fig. S4).

### Morality ratios of normal and aberrantly spliced transcripts for the deep intronic variants

For the deep intronic variants (nos. 3 and 7–9), the morality ratios of the normal and aberrantly spliced transcripts were determined using the 2100 Bioanalyzer Expert Software. The morality ratios differed between the HEK293T and HeLa cells in several variants as well as in the wild-type sample (Supplementary Table S4).

## Discussion

In this study, we demonstrated that all reported non-canonical splice site variants in *GLA* were associated with aberrant splicing. As the pathogenicity of non-canonical splice variants is quite complex to interpret, all candidate variants in this study were classified as having uncertain significance according to the ACMG guidelines. Furthermore, in silico tools often cannot predict the possibility of aberrant splicing in intronic variants that do not involve canonical splice sites. Therefore, validating splicing effects with an *in vitro* splicing assay is useful for confirming pathogenicity and determining associations with clinical phenotypes.

Patients with nonsense canonical splice sites, or frameshift variants, exhibit protein-truncating defects and generally show a classic phenotype [22]. Variant nos. 1 and 2 generated transcripts that had partial deletions in adjacent exons, resulting in protein-truncating variants. Although the study reporting variant no. 1 did not describe a phenotype, it was known that the male patient harboring variant no. 2 showed a classical phenotype [23]. Variant nos. 4–6 are located near exon 4, and the minigene assay revealed that they all exhibited complete exon 4 (92 bp) skipping. Patients harboring variant nos. 4 and 6 had classic phenotypes [24, 25], whereas the patient with variant no. 5 exhibited a later-onset phenotype [26]; however, this report did not specify sex, and thus it cannot be determined whether this was a heterozygous female or hemizygous male patient.

The minigene assay for variant no. 10 revealed whole exon 5 (162 bp) skipping, resulting in an in-frame deletion; furthermore, patients with variant no. 10 reportedly showed a classic phenotype [27]. Riera et al. investigated *GLA* sequence conservation patterns and revealed that amino acid residues 253–271, located near the active site, were highly conserved regions among multiple species [28]. Notably, variant no. 10 caused the deletion of residues 214–267, which may explain the classic phenotype. Variant no. 11 generated a transcript with 2 bp of intron 4 included, resulting in a frameshift mutation, another reasonable explanation for the classic phenotype [29]. The minigene assay for variant nos. 12 and 13 led to partial inclusion (36 bp) of intron 5, which initially appeared to be an in-frame insertion but was later found to create a stop codon in the fourth amino acid downstream of exon 5. Therefore, patients with these variants reportedly exhibited the classic phenotype [30]. For variant no. 12, the minigene assay results differed from those of the RT-PCR using the patients’ mRNA, possibly because of the hybrid minigene construction. Recent research indicates that splicing regulatory elements are typically within 200–300 nucleotides upstream and/or downstream of the regulated exon [31]. Therefore, considering intron 5 is a short intron, 140 nucleotides downstream of exon 5 could have been included when making the fragment in this study.

Variant nos. 3 and 7–9 were deep intronic variants that created cryptic exons, which were in the vicinity of the variants. These variants caused protein-truncating defects; the cryptic exon resulting from variant no. 3 consisted of 115 bp nucleotides, leading to frameshift insertion, whereas those resulting from variant nos. 7–9 contained premature-termination codons. The clinical phenotype, however, differed between the variants. Patients with variant nos. 3 and 7 exhibited the classic type [32, 33], while variant no. 9 caused a milder cardiac phenotype [34]. The direct RNA sequence of variant no. 9 revealed that the cryptic exon inclusion ratio (PSI) was 0.42, suggesting that approximately 60% of the transcripts were still normally spliced. This generation of normal and abnormal transcripts may, thus, reduce disease severity [35]. In addition, the morality ratios of normal and aberrantly spliced transcripts for variant nos. 3 and 7–9 differed from those in HEK293T and HeLa cells. Moreover, different cell types have different αGal A activity cutoffs [36]. Dai et al. [37] reported another deep intronic variant (c.639+1326C>T) detected in a male patient with Fabry disease who had the renal phenotype with a mild αGal A deficiency (75% of normal control). The mRNA extracted from his peripheral blood lymphocytes was subjected to RT-PCR, which revealed normal transcripts and two types of abnormally spliced transcripts (both were truncating types). The aberrantly spliced transcripts encoded deficient αGal A with about 25% of wild-type αGal A activity. The high residual αGal A activity was considered to be caused by normal transcript expression (a quarter of the total transcripts) and activity level of αGal A encoded by the abnormally spliced transcripts.

Notably, variant no. 9, which causes the cardiac type, has a high prevalence, especially in Taiwan (1 in 2810 people) [38]. Interestingly, Chiang et al. reported that the rates of variant carriers did not differ among healthy controls, people with type 2 diabetes, and people with cardiac disease in Taiwan [39]. A minigene assay was conducted in seven types of human cell lines, which revealed that variant no. 9 influenced alternative splicing in a tissue-specific manner [39]. Recent studies also found modulation of alternative splicing using antisense RNAs [40] and amiloride [41]. Thus, it is important to correctly identify mutations that cause aberrant splicing and verify the underlying mechanisms. Moreover, minigene analysis of single-nucleotide substitutions in introns with high allele frequencies, which were considered SNPs, showed normal splicing. This further confirms that minigene analysis correctly determines splicing abnormalities.

This study had several limitations. First, except for variant no. 9, the validated variants were not from our cohort but rather from reported cases. These are often less descriptive regarding clinical data, such as the Mainz Severity Score Index, which is widely used to indicate the clinical severity of Fabry disease [42]. Second, *in vivo* validation using patient samples was insufficient other than that for no. 9. Finally, as the hybrid plasmids used for the minigene splicing assay were constructed artificially, the assay did not always imitate the *in vivo* splicing reaction. However, several previous studies performed transcriptional analyses via RT-PCR using patient mRNA, which mostly produced results consistent with ours [32–34].

In conclusion, our study revealed and confirmed splicing defects in 13 variants of the *GLA* gene. This analysis of splicing aberrations established correlations with pathogenicity and may contribute to elucidating tissue-specific splicing mechanisms.

## Supplementary Information

Below is the link to the electronic supplementary material.

Supplementary file1 (DOCX 7004 kb)

## Data Availability

The data are not publicly available to maintain the confidentiality of patients’ details but can be obtained from the corresponding author upon reasonable request.
