# Supplementary material for: All reported non-canonical splice site variants in GLA cause aberrant splicing
Source: Clin Exp Nephrol. 2023 May 31;27(9):737–46. doi: 10.1007/s10157-023-02361-x (PMC10432374; doi:10.1007/s10157-023-02361-x)
Supplement: Supplementary file 1 — Supplementary file1 (DOCX 7004 kb) [file 10157_2023_2361_MOESM1_ESM.docx]

Supplementary materials

**All reported non-canonical splice site variants in *GLA* cause aberrant splicing**

Contents

**Supplementary Table S1.** Primer sequences

**Supplementary Figure S1.** Overlap PCR for Plasmid construction (No.7-9)

**Supplementary Table S2.** Interpretation of pathogenicity of the variants

**Supplementary Table S3.** In-silico prediction for splicing defects

**Supplementary Figure S2.** Sequences of RT-PCR product generated from minigene assay

**Supplementary Figure S3.** Result of minigene assay for intronic variant with high allele frequency

**Supplementary Figure S4.** Caluculation of cryptic exon inclusion ratio

**Supplementary Table S4.** Morality ratio of normal transcript and aberrantly spliced transcript for deep intronic variants (No.3 and 7-9)

**Supplementary Table S1.** Primer sequences

**Supplementary Figure S1.** Overlap PCR for Plasmid construction (No.7-9)


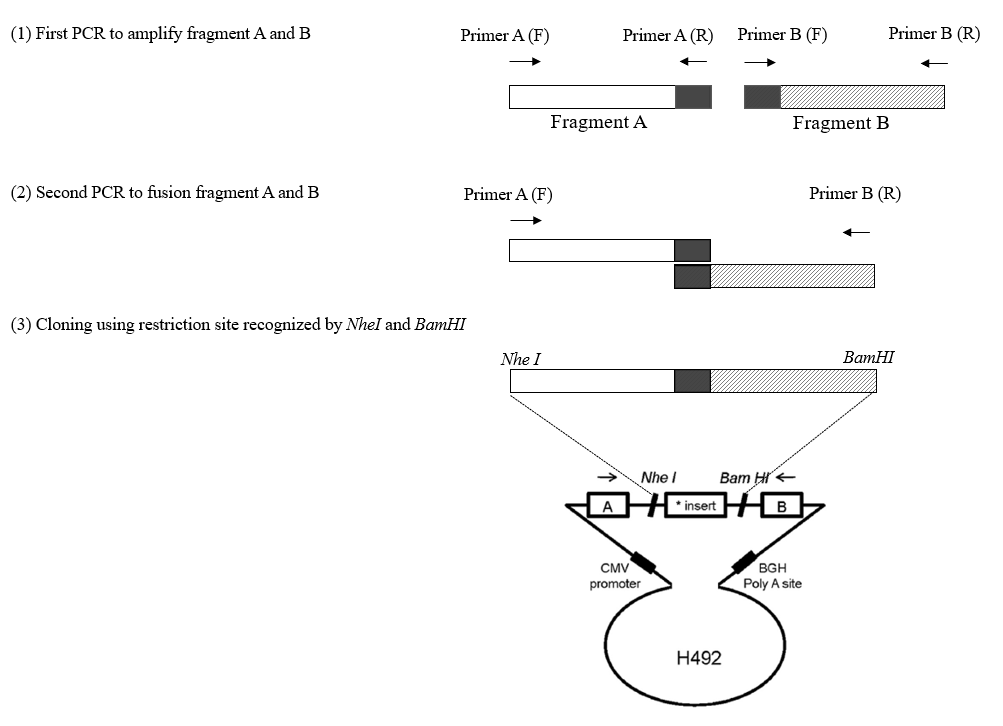


**Supplementary Table S2.** Interpretation of pathogenicity of the variants

*Referenced population databases are gnomAD (<https://gnomad.broadinstitute.org/>), 1000G (<https://www.internationalgenome.org/>) and HGVD (https://www.hgvd.genome.med.kyoto-u.ac.jp/download.html).

**Supplementary Table S3.** *In-silico* prediction for splicing defects

DS, splice donor site; AS, splice acceptor site; WT, wild type; MT, mutant; ESS, exonic splicing silencer; ESE, exonic splicing enhancer.

Bold figures met the prediction criteria described in the Methods section.

**Supplementary Figure S2.** Sequences of RT-PCR product generated from minigene assay

(A)


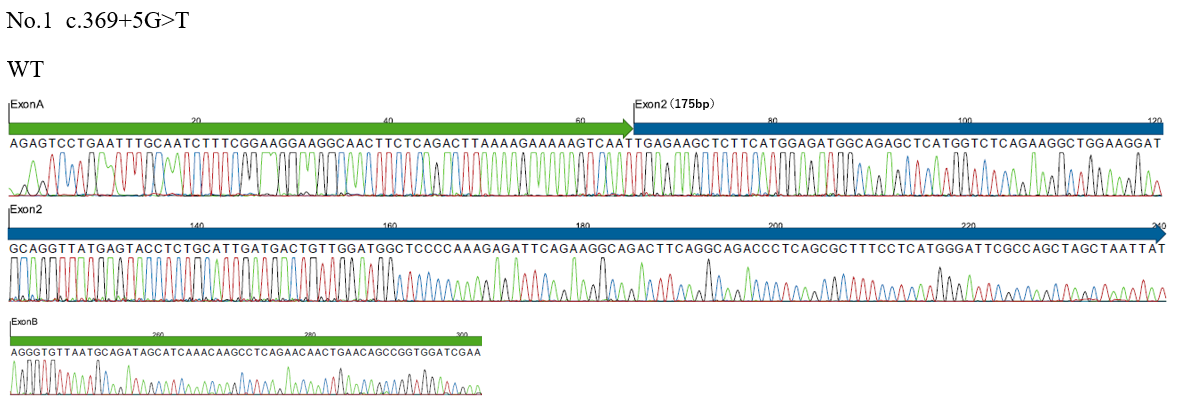


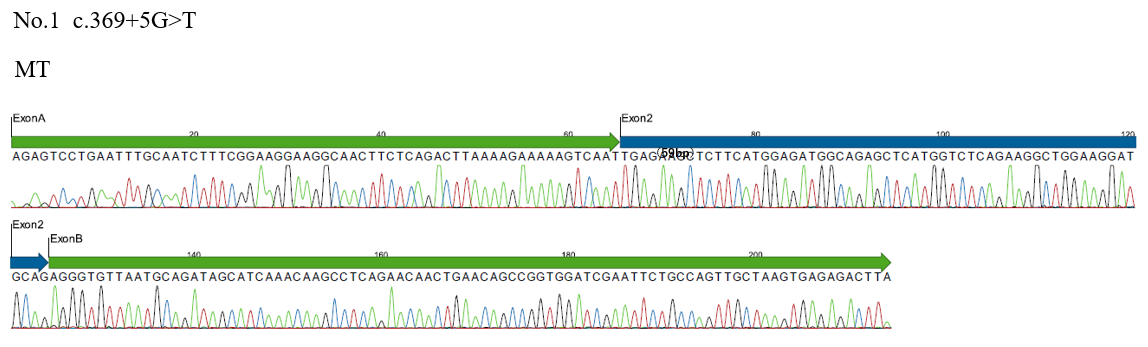


(B)


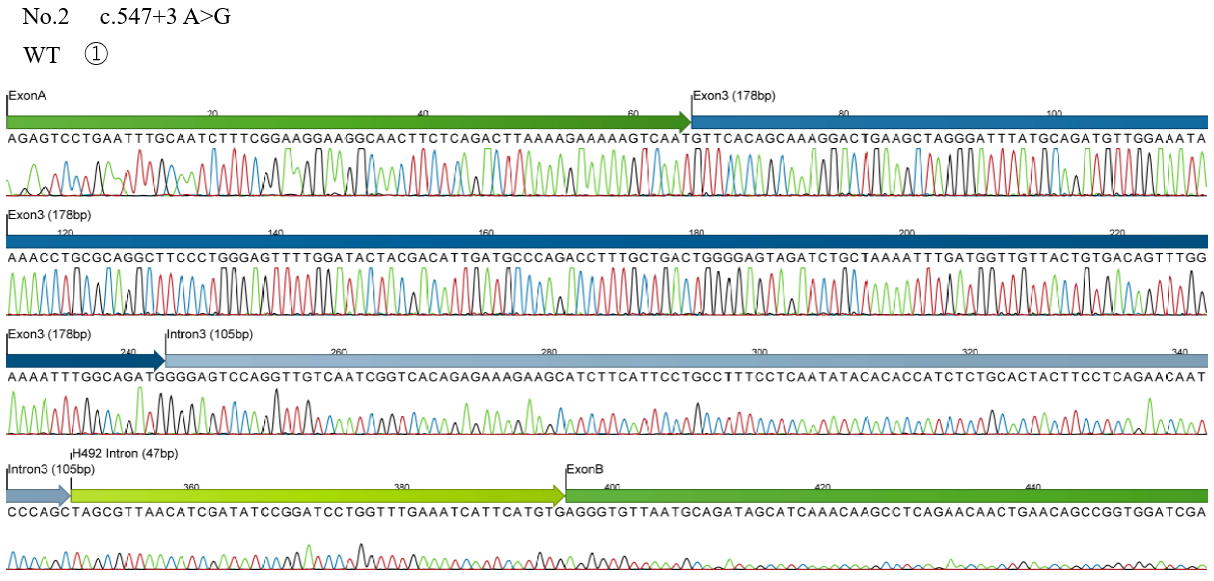


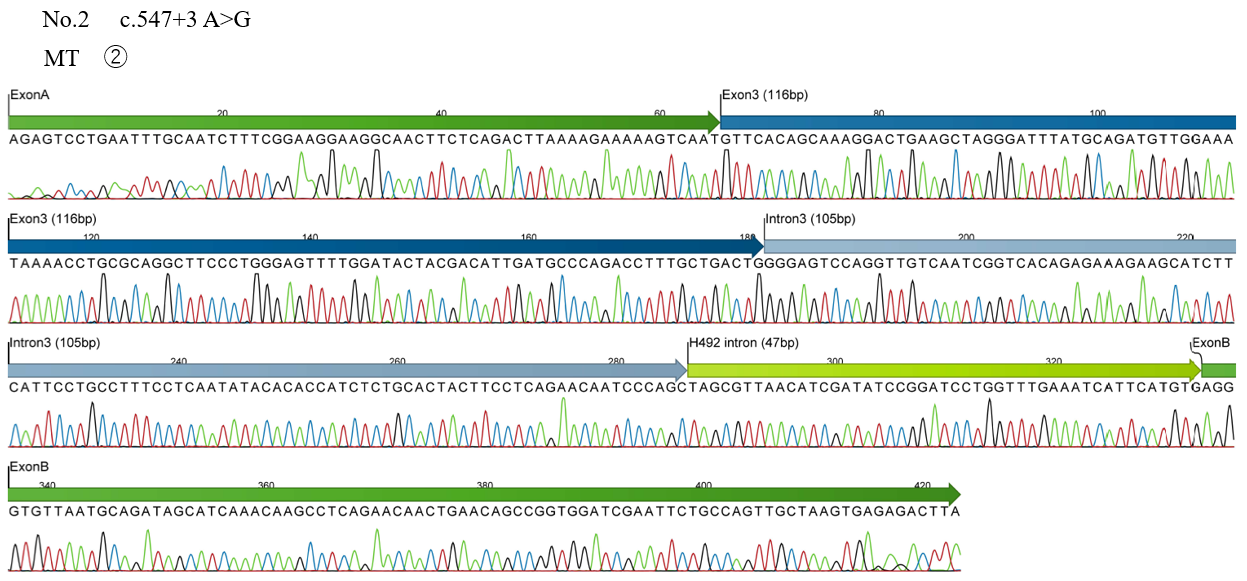


(C)


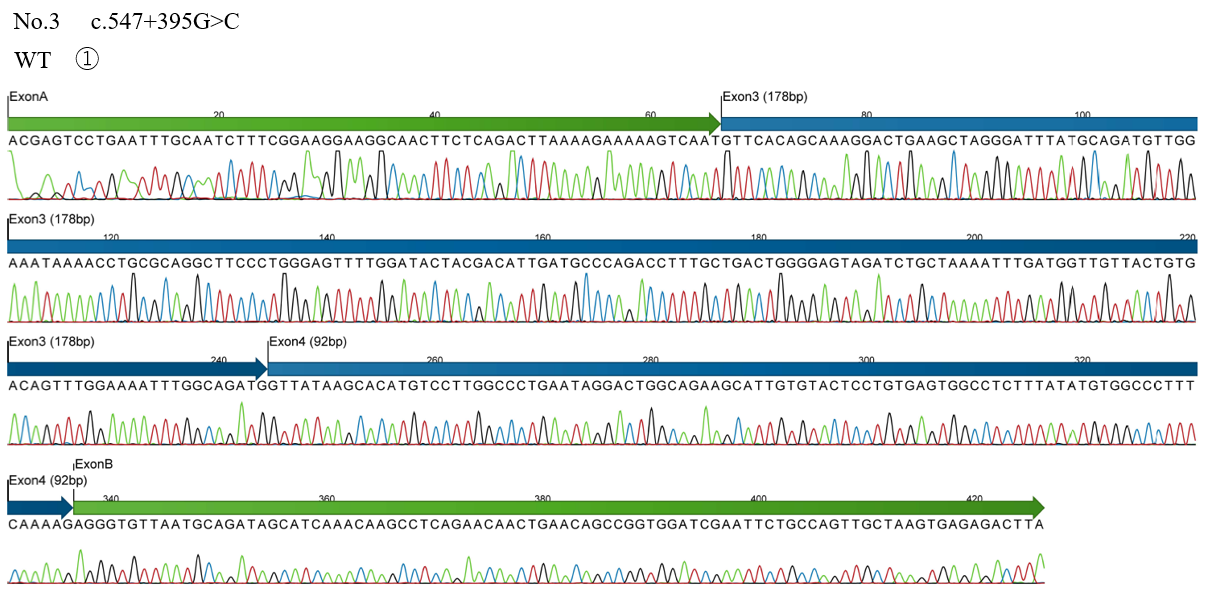


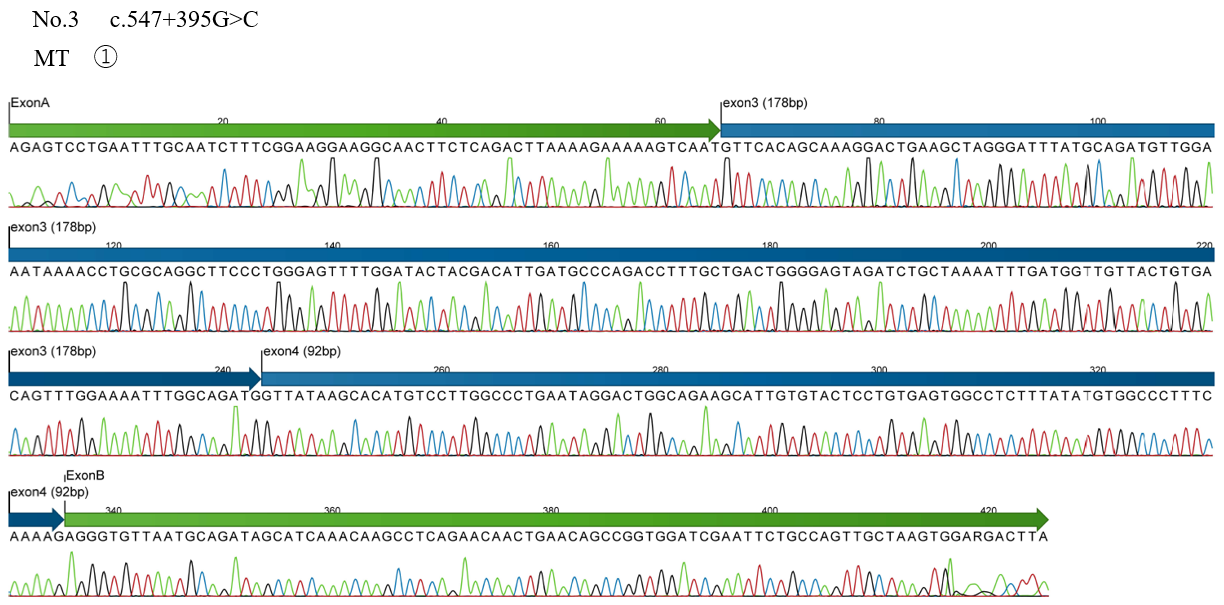


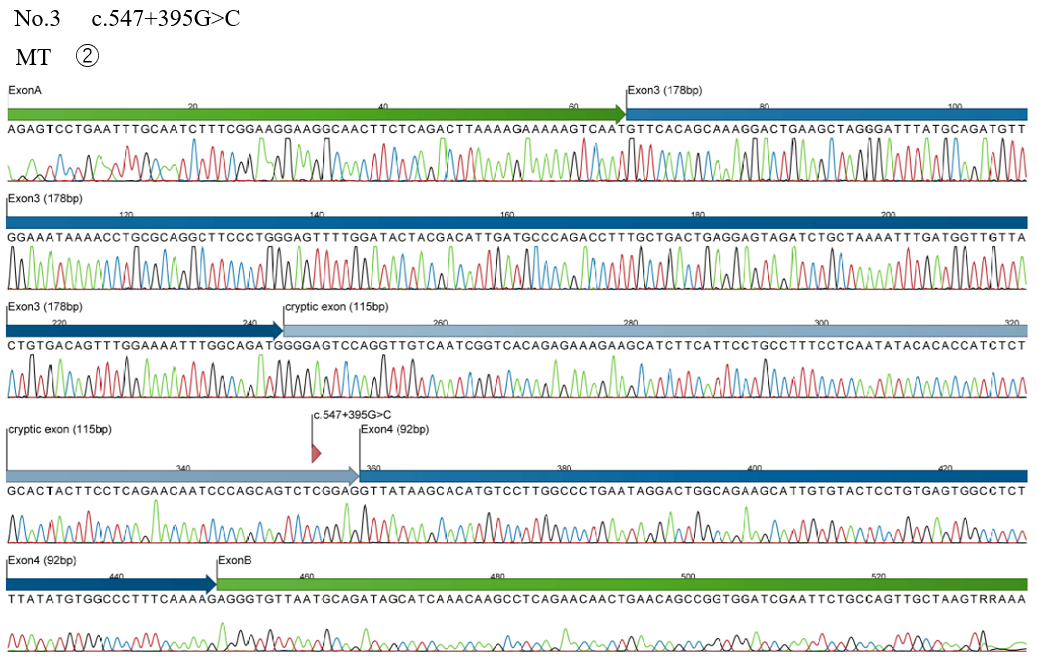


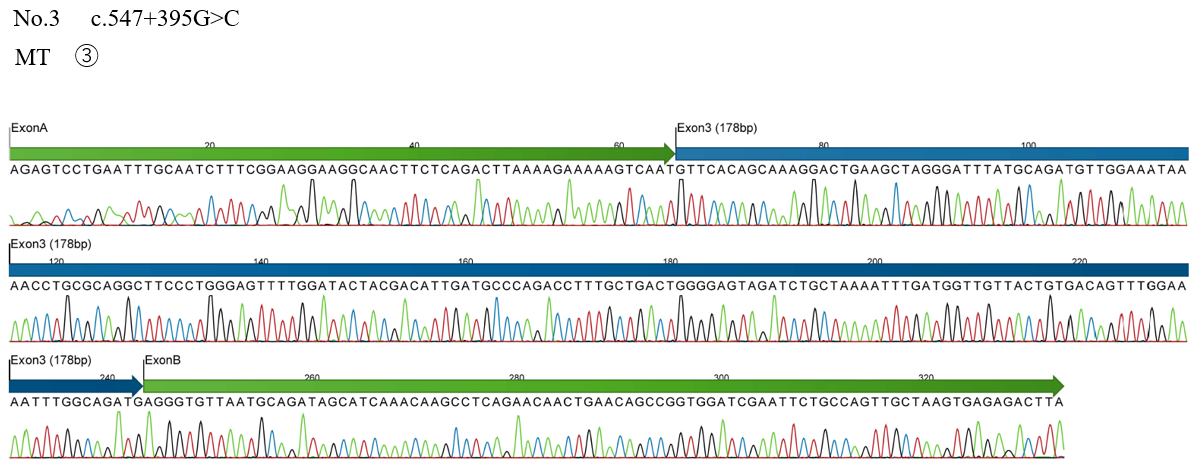


(D)


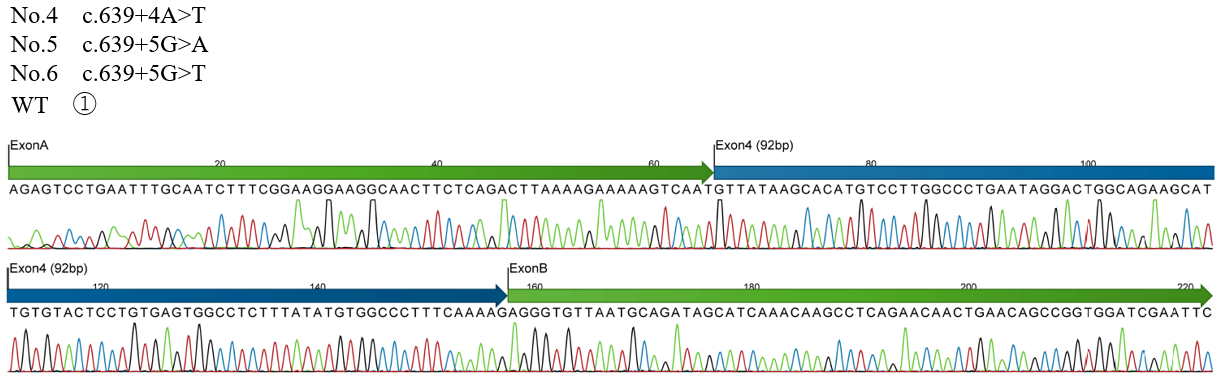


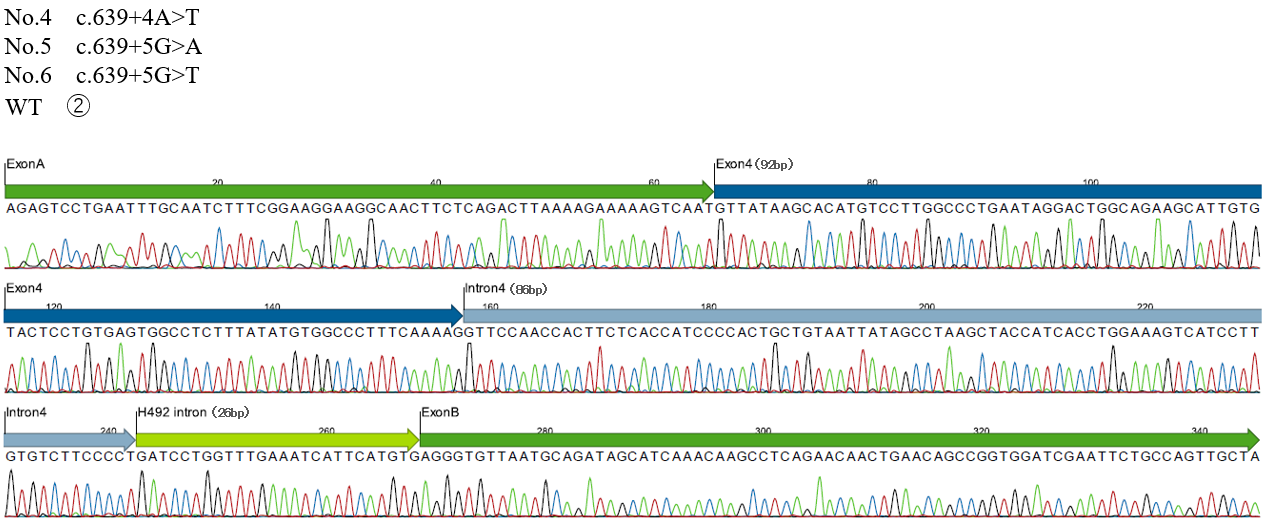


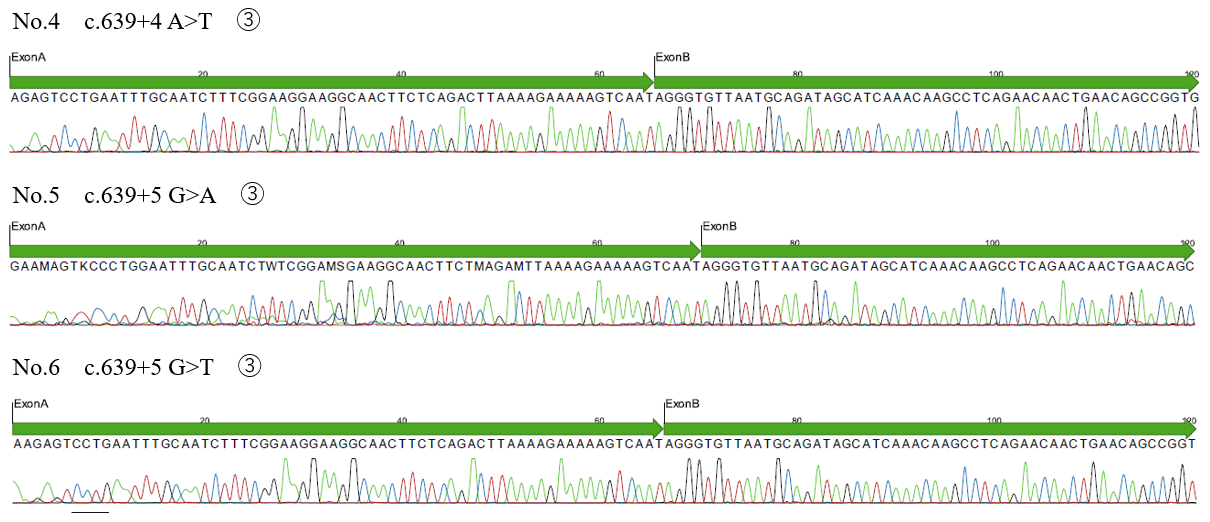


(E)


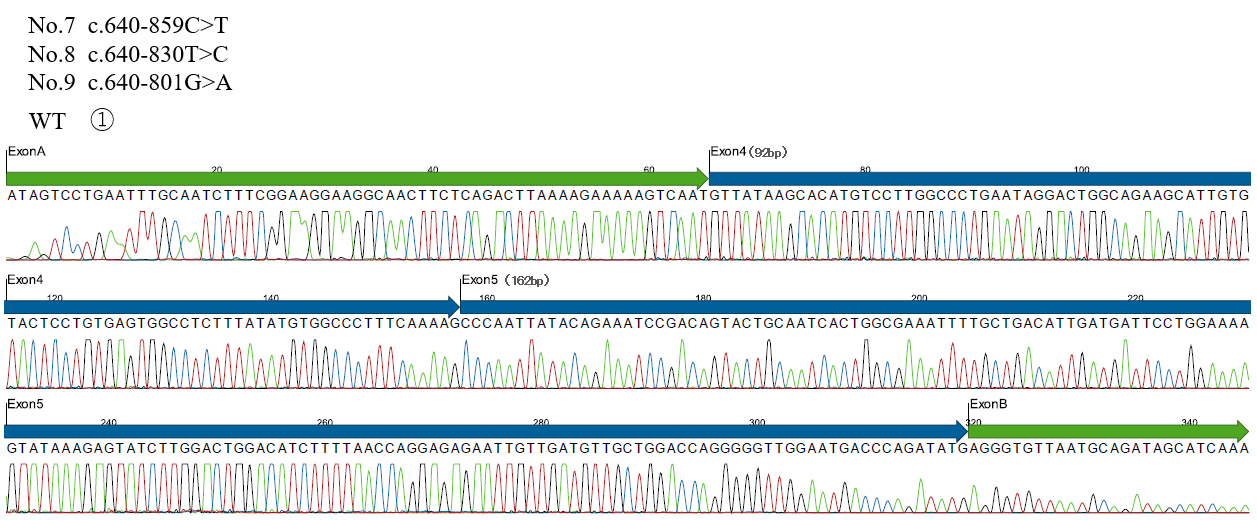


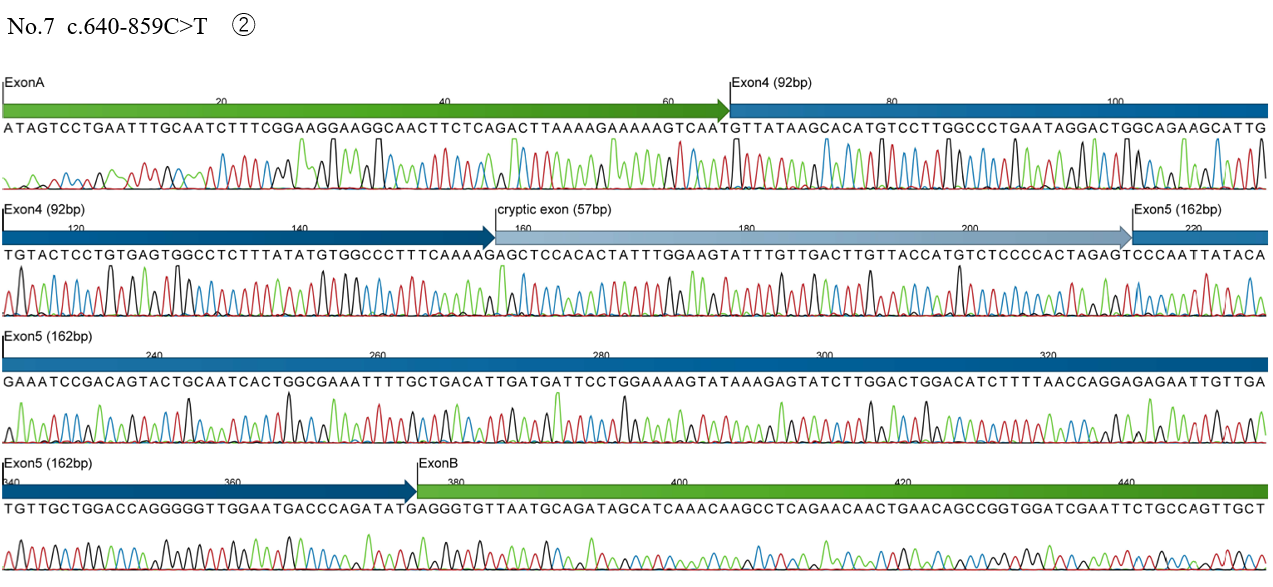


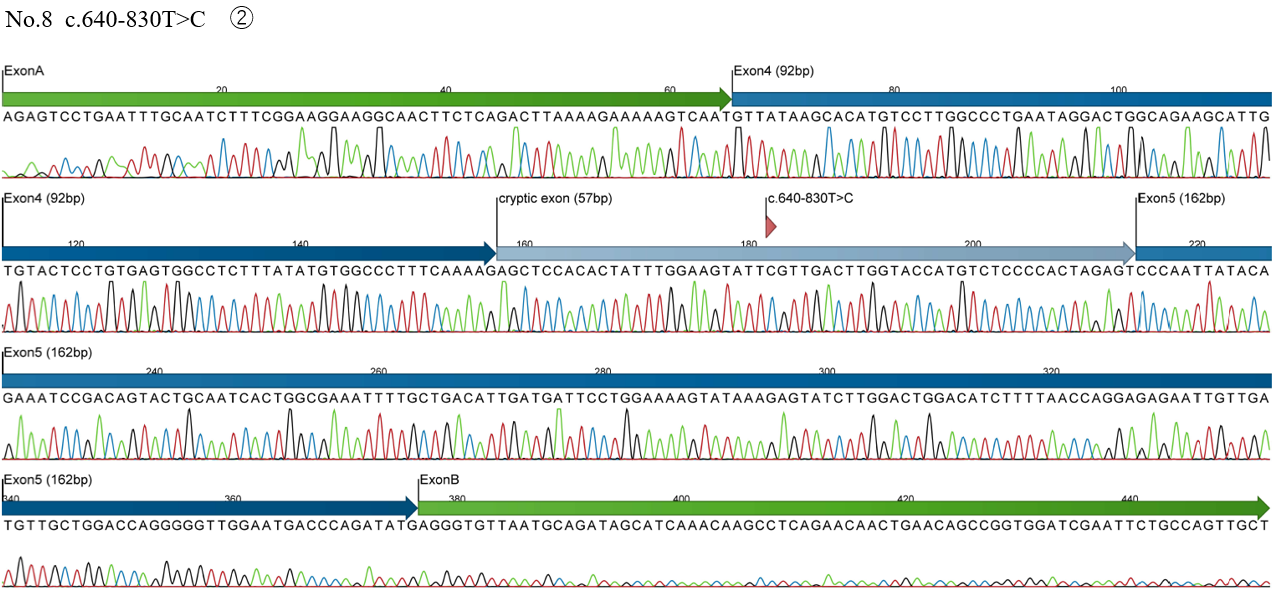


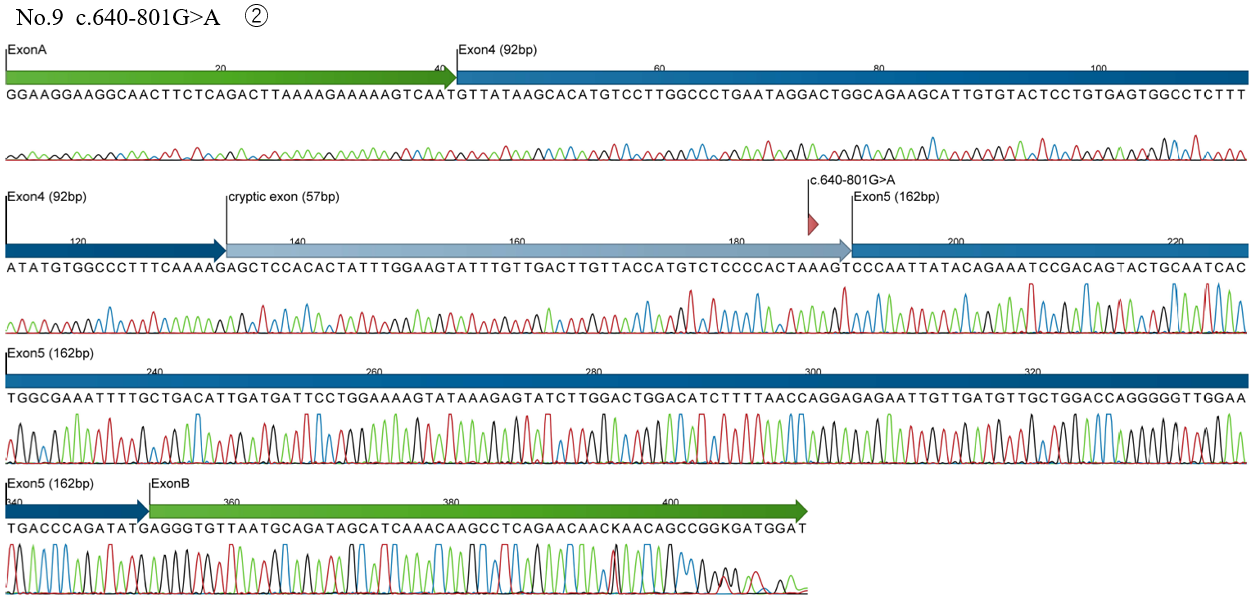


(F)


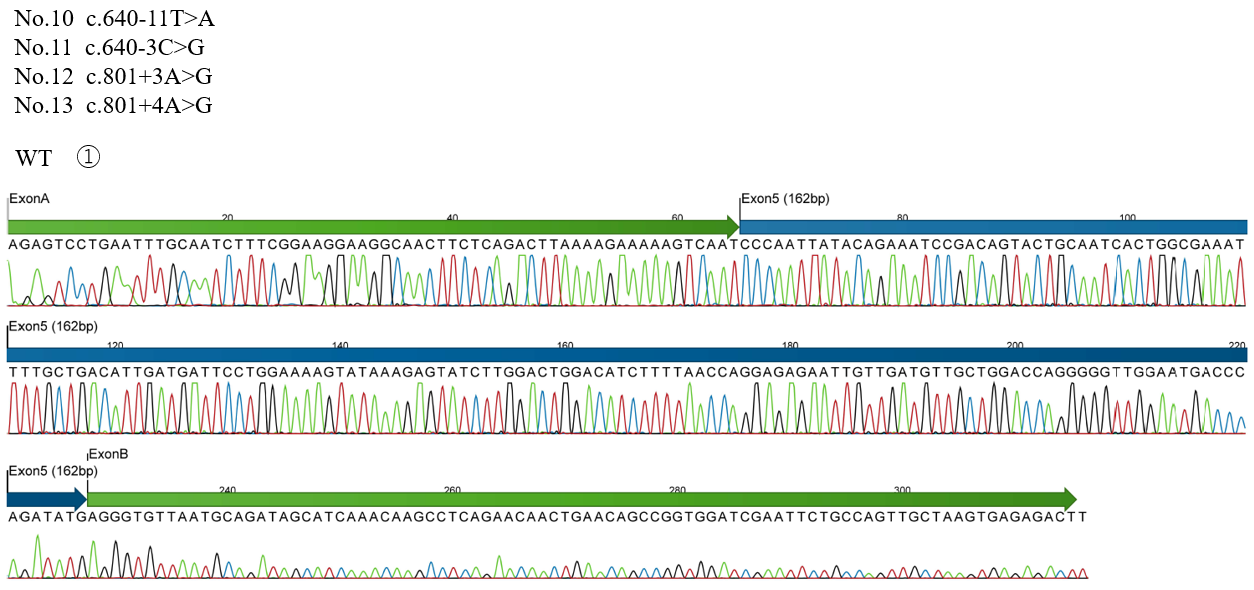


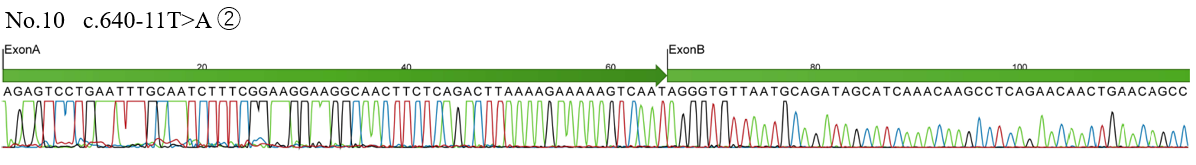


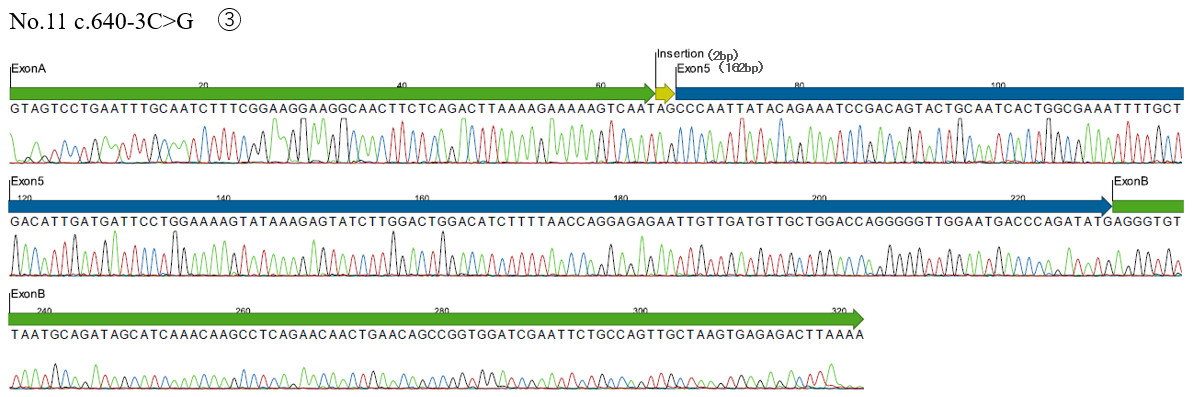


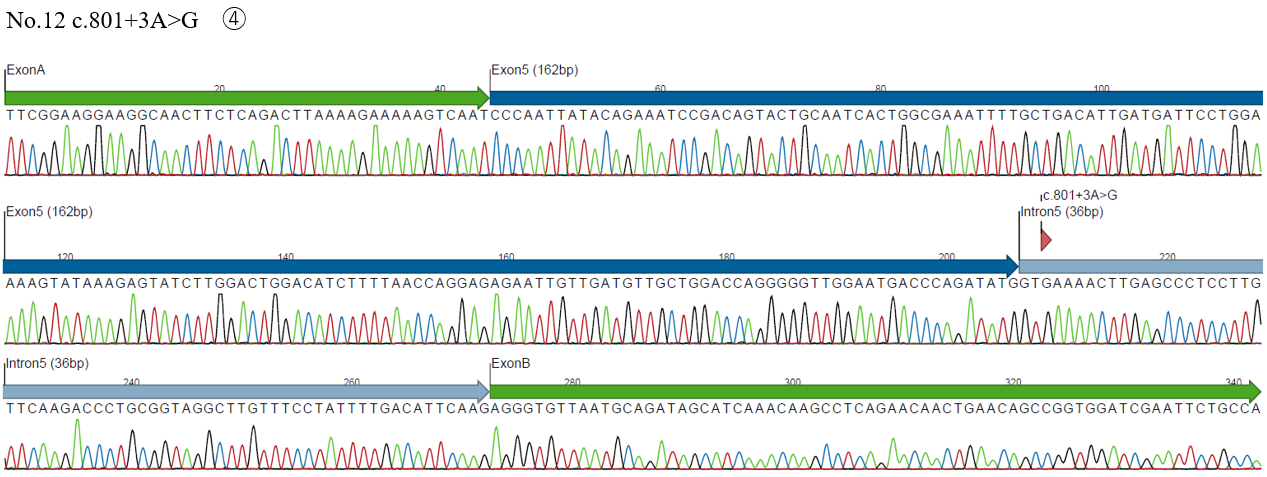


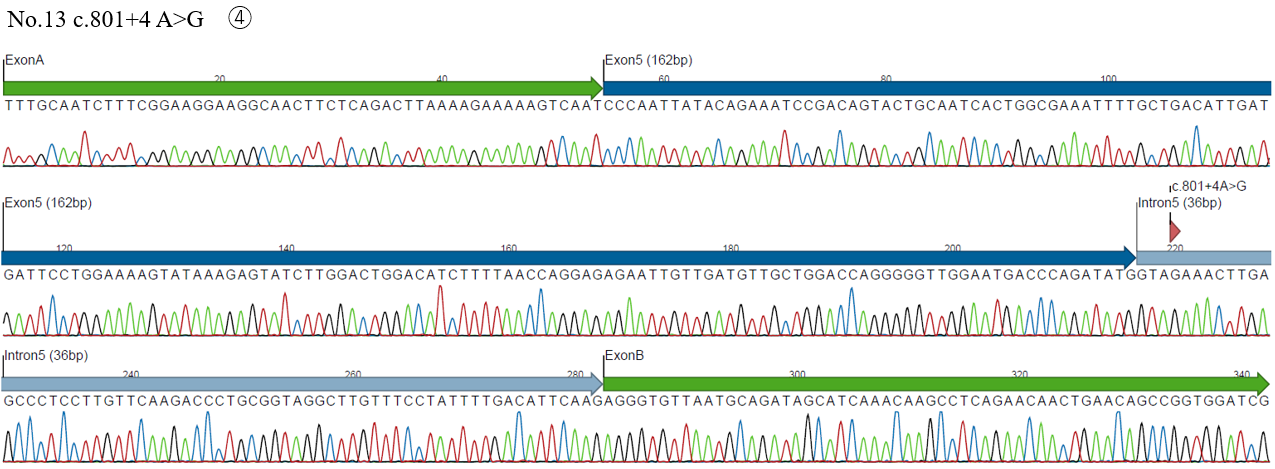


**Supplementary Figure S3.** Result of minigene assay for intronic variant with high allele frequency

（A）


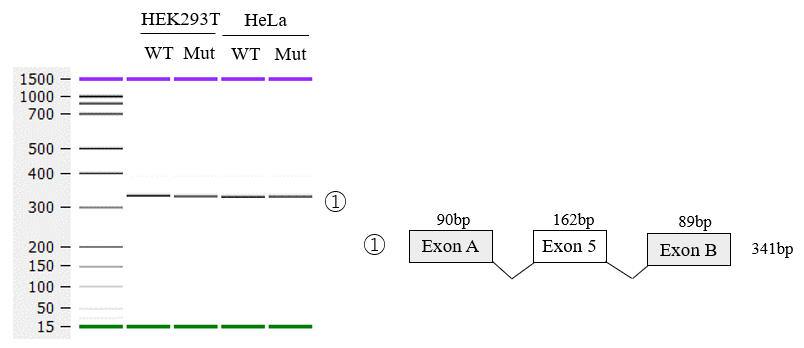


(B)WT


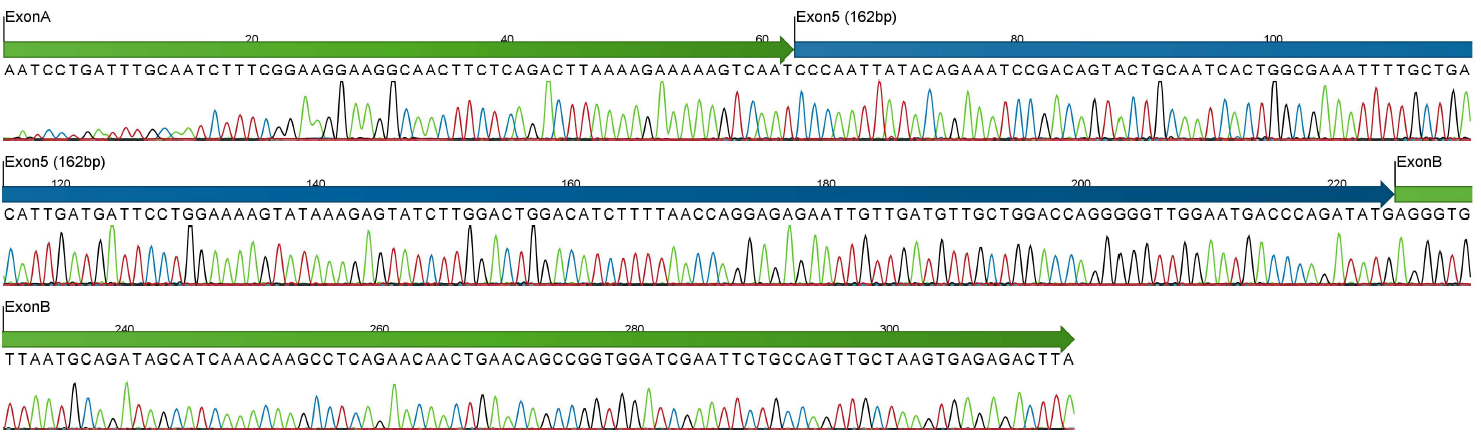


(C)mutant


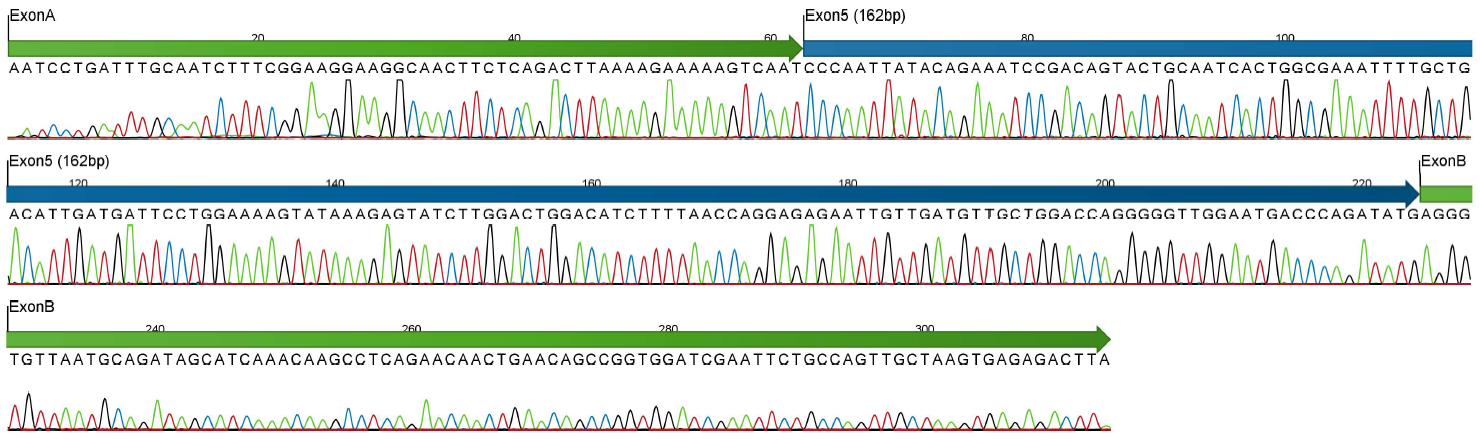


**Supplementary Figure S4.** Caluculation of cryptic exon inclusion ratio

Cryptic exon inclusion ratio, known as percent spliced in (PSI), was calculated using the follwing formula

PSI = (Ji/2) / (Ji/2 + Js)

Ji is the number of inclusion junction reads aligned to upstream and downstream splice junctions of cryptic exon. Ji is to be devided by 2 because the are two junctions for an exon inclusion.

Js is the number of reads aligned to the junction that skips the cryptic exon. Values of PSI can range from 0 (completely skipped) to 1 (complete inclusion).


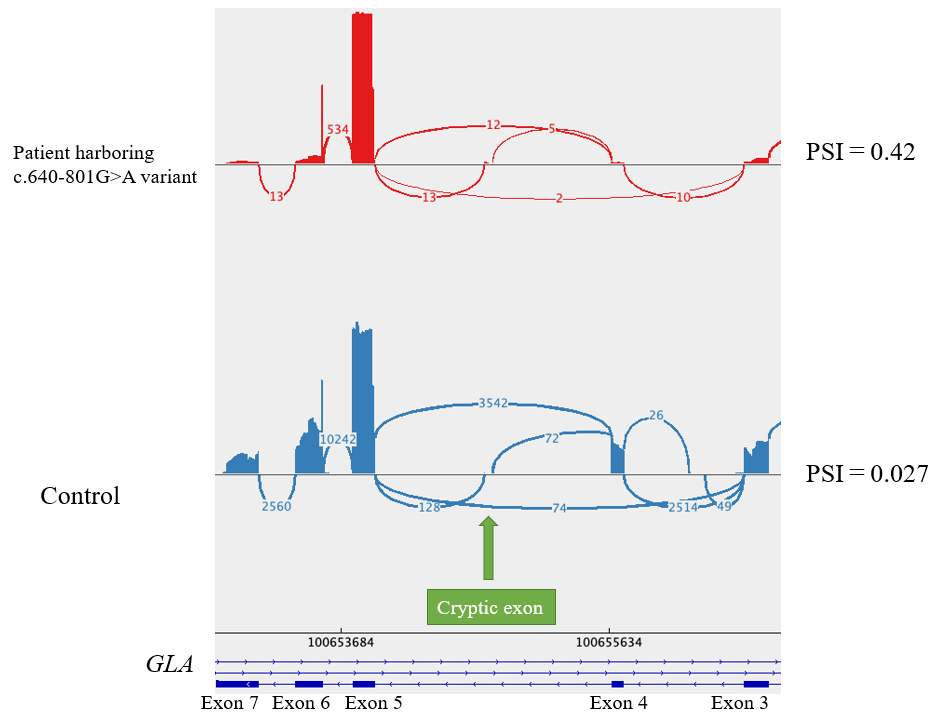


**Supplementary Table S4.** Morality ratio of normal transcript and aberrantly spliced transcript for deep intronic variants (No.3 and 7-9)

| Variant No. | Wild type（WT）  / variant | Cell type | Morality (nmol/L) | | Morality ratio (B/A) |
| --- | --- | --- | --- | --- | --- |
|  |  |  | Normal transcript (A) | Transcript containing cryptic exon (B) |  |
| 3 | WT | HEK | 14.1 | Not detectable | ≒0 |
|  |  | HeLa | 14.4 | 1.4 | 0.1 |
|  | 3 | HEK | 3.4 | 3.4 | 1.0 |
|  |  | HeLa | 3.8 | 2.6 | 0.7 |
| 7-9 | WT | HEK | 56.6 | 6.3 | 0.1 |
|  |  | HeLa | 41.3 | 3.1 | 0.1 |
|  | 7 | HEK | 41.5 | 14.9 | 0.4 |
|  |  | HeLa | Not detectable | 46.6 | - * |
|  | 8 | HEK | 14.2 | 10.8 | 0.8 |
|  |  | HeLa | 21.5 | 20 | 0.9 |
|  | 9 | HEK | Not detectable | 57.2 | - * |
|  |  | HeLa | 18.1 | 31 | 1.7 |

*, incalculable because denominator is not detectable (close to 0)
